# Hypothermia and Rewarming Induce Gene Expression and Multiplication of Cells in Healthy Rat Prostate Tissue

**DOI:** 10.1371/journal.pone.0127854

**Published:** 2015-05-21

**Authors:** Helena Kaija, Lasse Pakanen, Marja-Leena Kortelainen, Katja Porvari

**Affiliations:** 1 Department of Forensic Medicine, Faculty of Medicine, University of Oulu, Oulu, Finland; 2 Medical Research Center Oulu, Oulu University Hospital and University of Oulu, Oulu, Finland; University of Catania, ITALY

## Abstract

Prostate cancer has been extensively studied, but cellular stress responses in healthy prostate tissue are rarely investigated. Hypothermia is known to cause alterations in mRNA and protein expressions and stability. The aim of this study was to use normal rat prostate as a model in order to find out consequences of cold exposure and rewarming on the expressions of genes which are either members or functionally/structurally related to erythroblastic leukemia viral oncogene B (ErbB) signaling pathway. Relative mRNA expressions of *amphiregulin* (*AMR*), *cyclin D1* (*CyD1*), *cyclin-dependent kinase inhibitor 1A* (*p21*), *transmembrane form of the prostatic acid phosphatase* (*PAcP*), *thrombomodulin* (*TM*) and *heat shock transcription factor 1* (*HSF1*) in rat ventral prostate were quantified in mild (2 or 4.5 h at room temperature) and severe (2 or 4.5 h at +10°C) hypothermia and in rewarming after cold exposure (2 h at +10°C followed by 2 h at room temperature or 3 h at +28°C). AMR protein level, apoptotic *Bcl-2 associated X protein to B-cell CLL/lymphoma 2 *(*Bax*/*Bcl-2*) mRNA ratio and proliferative index Ki-67 were determined. 4.5-h mild hypothermia, 2-h severe hypothermia and rewarming increased expression of all these genes. Elevated proliferation index Ki-67 could be seen in 2-h severe hypothermia, and the proliferation index had its highest value in longer rewarming with totally recovered normal body temperature. Pro-apoptotic tendency could be seen in 2-h mild hypothermia while anti-apoptosis was predominant in 4.5-h mild hypothermia and in shorter rewarming with only partly recovered body temperature. Hypothermia and following rewarming promote the proliferation of cells in healthy rat prostate tissue possibly via ErbB signaling pathway.

## Introduction

Thermoregulatory mechanisms control core body temperature tightly in most mammals. Intentional induction of lower body temperature has been proved to protect some tissues, for example during surgery, and molecular level mechanisms behind the physiological processes are being characterized. Depending on the intensity of cold exposure, divergent changes in gene and protein expressions have been shown. In mild hypothermia a reduction in degradation of mRNAs and proteins, increased or decreased transcription of specific genes and increased or decreased synthesis of proteins has been reported during cold exposure and following rewarming *in vitro*. As temperature keeps dropping, general inhibition of transcription and translation occurs and eventually disruption of cellular structures follows [1, 2].

ErbB signaling pathway is involved in fundamental cellular processes regulating cell proliferation, survival and migration in a variety of tissues of epidermal origin. Epidermal growth factor receptor (EGFR) is a transmembrane tyrosine kinase, and a member of ErbB-family of four structurally related receptors. In mammalian cells seven EGFR-ligands have been identified. All of these ligands are synthetized as transmembrane proteins, consisting of conserved extracellular receptor-binding EGF-domain/domains, a transmembrane domain and a C-terminal intracellular domain. Soluble growth factors containing EGF-domain are cleaved by cell surface enzymes [3]. Ligand-induced activation of the receptor results in autophosphorylation and transphosphorylation within its intracellular domain, and leads to activation of the signaling pathway [4].

AMR is an EGFR-ligand, containing an additional amino-terminal heparin-binding domain [5]. AMR expression modulates branching and morphogenesis of normal tissues such as mammary gland, lung, kidney and prostate, and it is upregulated in numerous neoplasms [6]. In prostate, AMR is expressed in luminal secretory epithelium [7]. The expression of prostatic AMR protein increases progressively from benign epithelium to high-grade prostatic intraepithelial neoplasia and cancer [8]. In non-transformed prostate epithelial cells ADAM-family proteases are involved in regulation of AMR shedding and subsequent EGFR-activated promotion of cell proliferation. Transformed prostatic cells might use other mechanisms [3, 9].

Numerous other proteins possess EGF-like motifs, including TM, a transmembrane protein essentially involved in blood coagulation and inflammation [10, 11]. This group of proteins has very low receptor-binding affinity, but yet in some cases potent activity in signal transduction [12, 4].

Our previous study clarified the effects of hypothermia and rewarming on cardiac gene and protein expressions [13]. Most studies concerning prostate involve tissue abnormalities, such as inflammation, hyperplasia or malignancy. In development of cancer therapy, the ErbB pathway appears as an attractive target. There is, however, evidence that the divergent downstream pathways can also cause unwanted consequences [14]. In this study, we wanted to examine effects of cold exposure and rewarming on selected representative genes related to ErbB pathway and on some structurally or functionally similar genes in normal, healthy prostate tissue. The use of a rat model made it possible to study these effects in whole, living organism.

Anesthetized rats were exposed to different periods of different ambient temperatures with or without following rewarming. Ventral lobes of rat prostate tissues were used in determination of the relative mRNA expressions of *AMR*, *TM* and the transmembrane form of the *PAcP*, which down-regulates prostate cell growth in human prostate cancer cells [15]. The relative mRNA expressions of *p21* and *CyD1*, regulators of cell cycle progression [16, 17] as well as anti-apoptotic *Bcl-2* and pro-apoptotic *Bax*, both engaged in EGF-induced apoptosis [18] were quantified. HSFs are known to coordinate transcription in response to stress, including thermal stress [19], and therefore we also determined the mRNA expression level of *HSF1*. The protein levels of cellular AMR and assessment of the proliferation rates of cells by calculating Ki-67 proliferative index in rat prostate tissues were done using immunohistochemistry. Correlations between the variables were studied. Our results suggest that ErbB pathway is activated in hypothermia and following rewarming in rat prostate tissue.

## Materials and Methods

### Ethical statement

96 Sprague-Dawley male rats (*Rattus norvegicus*) aged between 8 and 9 weeks and weighing an average of 329 g were used in the study. The rats were reared in the Laboratory Animal Centre (license no: PSAVI-2010-01204/Ym-23), University of Oulu, Finland. All experiments were conducted according to the protocol approved by the Finnish National Animal Experiment Board (license no: ESAVI/974/04.10.03/2012). The research plan was approved by the Northern Ostrobothnia Hospital research ethics committee (no: 31/2007).

### Experimental design

As the license required the animals were under anesthesia during cold exposure. The experimental design, animal welfare and the methods are described in detail in our article published earlier [13]. Briefly: anesthetized (fentanyl/fluanisone/midazolam) rats were randomly divided into seven groups. C, control group: the rats were killed immediately; MH1, mild hypothermia 1: the rats were kept at room temperature (+21 ± 2°C) for 2 hours; MH2, mild hypothermia 2: the rats were kept at room temperature for 4.5 hours; SH1, severe hypothermia 1: the rats were kept at +10°C for 2 hours; SH2, severe hypothermia 2: the rats were kept at +10°C until their rectal temperatures had decreased to +20°C with a 4.5-hour mean duration of cold exposure; SHW1, severe hypothermia followed by rewarming at room temperature: the rats were kept at +10°C for 2 hours, and then at room temperature for 2 hours; SHW2, severe hypothermia followed by rewarming at +28°C: the rats were kept at +10°C for 2 hours, and after that they were moved into an incubator with a temperature of +28°C for 3 hours. During the last 3 hours, no anesthetic was given to the rats in SHW2 group to ensure recovery of the normal body temperature. The rectal temperature data were collected at intervals of 5 minutes except for the rats in the SHW1 and SHW2 groups, in which the temperature was recorded only at the beginning of the test, at the 2-hour interval and at the end of the test. MH1 group consisted of six rats, while all the other groups consisted of 15 rats.

The mean rectal endpoint temperatures of the rats were: C, 37.2 ± 0.1°C; MH1, 30.3 ± 0.4°C; MH2, 30.0 ± 0.2°C; SH1, 23.8 ± 0.4°C; SH2, 20.0 ± 0.0°C; SHW1, 28.4 ± 0.5°C and SHW2, 37.4 ± 0.2°C.

The rats in groups MH1 and MH2 were not warmed during the experiments allowing us to monitor the anesthesia-induced decrease in body temperature, which cannot be avoided by external warming [20, 21]. Thus MH1 group serves as a control for SH1 group and MH2 group for SH2 group. Anesthesia combined to cold exposure decreased the mean body temperatures of rats faster than anesthesia alone, leading to significantly lower endpoint temperatures: MH1 vs. SH1, *p* = 0.000; MH2 vs. SH2, *p* = 0.000 [13].

### Real-time quantitative RT-PCR

Total RNA was extracted from rat prostate ventral lobe tissues stored at -80°C with a miRNeasy mini kit (Qiagen, Hilden, Germany) using an automated QIAcube sample preparation instrument (Qiagen, Hilden, Germany) according to the manufacturer’s protocols. A High Capacity cDNA RT kit (Applied Biosystems, Foster City, CA) was used to reverse transcribe the RNAs with random primers according to the manufacturer’s protocol. The complementary DNAs were amplified in duplicate with a Rotor-Gene Q (Qiagen, Hilden, Germany) using gene-specific primers (Sigma, Haverhill, UK). Sequences of primers and amplicon sizes are listed in Table 1. The mean fold change in relative expression of target gene at each group was calculated using 2^-∆∆Ct^ method and *GAPDH* as the reference gene.

**Table 1:** Primers used in qPCR analysis.

Gene symbol	Direction	Primer sequence	Amplicon size
*AMR*	Forward	GTGCATGCCATTGCCTAGCTGA	78
	Reverse	TCATTTCCGGTGTGGCTTGGCA	
*Bax*	Forward	CCAGGACGCATCCACCAAGAAGC	136
	Reverse	TGCCACACGGAAGAAGACCTCTCG	
*Bcl-2*	Forward	GAGGCTGGGATGCCTTTGTGGA	89
	Reverse	GCTGAGCAGCGTCTTCAGAGA	
*CyD1*	Forward	ATCAAGTGTGACCCGGACTG	216
	Reverse	GCCACTACTTGGTGACTCCC	
*HSF1*	Forward	CCATGAAGCACGAGAACGAG	117
	Reverse	ACTGCACCAGTGAGATCAGGA	
*PAcP*	Forward	CGGGATCCTGGTGATATTGCT	70
	Reverse	CCGATACACGTCTCTCTGCC	
*p21*	Forward	ACATCTCAGGGCCGAAAACG	78
	Reverse	CTTGCAGAAGACCAATCGGC	
*TM*	Forward	GATCTCCATTGCCAGCCT	140
	Reverse	CACGTGCTGCAGTACTACCT	
*GAPDH*	Forward	TGGAAGGACTCATGACCACA	160
	Reverse	TTCAGGTCAGGGATGACCTT	

The relative expression of *Bax* mRNA to the relative expression of *Bcl-2* mRNA ratio of each ventral prostate tissue was calculated.

### Immunohistological analysis

A part of each prostate ventral lobe tissue was fixed with formalin, embedded in paraffin and sectioned at a thickness of 5 μm. Sample preparation was carried out by the PT-link system and an automated staining instrument with the EnVision detection system (DAKO, Glostrup, Denmark). Mouse monoclonal Amphiregulin antibody sc-74501 (Santa Cruz Biotechnology, Santa Cruz, CA, USA) at 1:400 dilution and mouse monoclonal anti-rat Ki-67 antigen, clone MIB5, M7248 (DAKO, Glostrup, Denmark) at 1:75 dilution were used to detect AMR and Ki-67 protein, respectively, in rat prostate ventral lobe sections. The slides were counterstained with hematoxylin (Ragena, Toivala, Finland), observed with a Nikon Microphot SA microscope (Nikon, Tokyo, Japan) and photographed with a Nikon FX35DX camera (Nikon, Tokyo, Japan). AMR immunoreactivity was divided into three categories: absent or weak staining intensity (1), moderate staining intensity (2) and strong staining intensity (3). Nuclear Ki-67 staining was defined as absent or present. Ki-67 index was calculated as percentage of Ki-67 positive nuclei of the total number of nuclei. 229 ± 12 nuclei of each slide were counted using ImageJ Cell Counter plugin (rsb.info.nih.gov/ij/).

### Statistics

Statistical analyses were conducted using IBM SPSS statistics, version 19 (Armonk, NY, USA). The pair-wise analyses between the groups were done either with the parametric t-test (statistical significance level set at *p* <0.05) or with the non-parametric Mann-Whitney test (statistical significance level set at *p* <0.05) appropriately. Correlations between the variables within the groups were evaluated by Pearson correlation test (two-tailed; statistical significance level set at *p* <0.05). The data are given as mean ± standard error of the mean (SEM).

Statistical comparisons were done between rational pairs of groups in order to compare two groups that differ either in the treatment temperature or in its duration. The pairs were: MH1 vs. MH2, SH1 vs. SH2, MH1 vs. SH1, MH2 vs. SH2, SH1 vs. SHW1 and SH1 vs. SHW2. Additional comparisons between C and the other groups were also done.

## Results

### Relative mRNA expressions in rat prostate ventral lobe tissue

**Fig 1:**
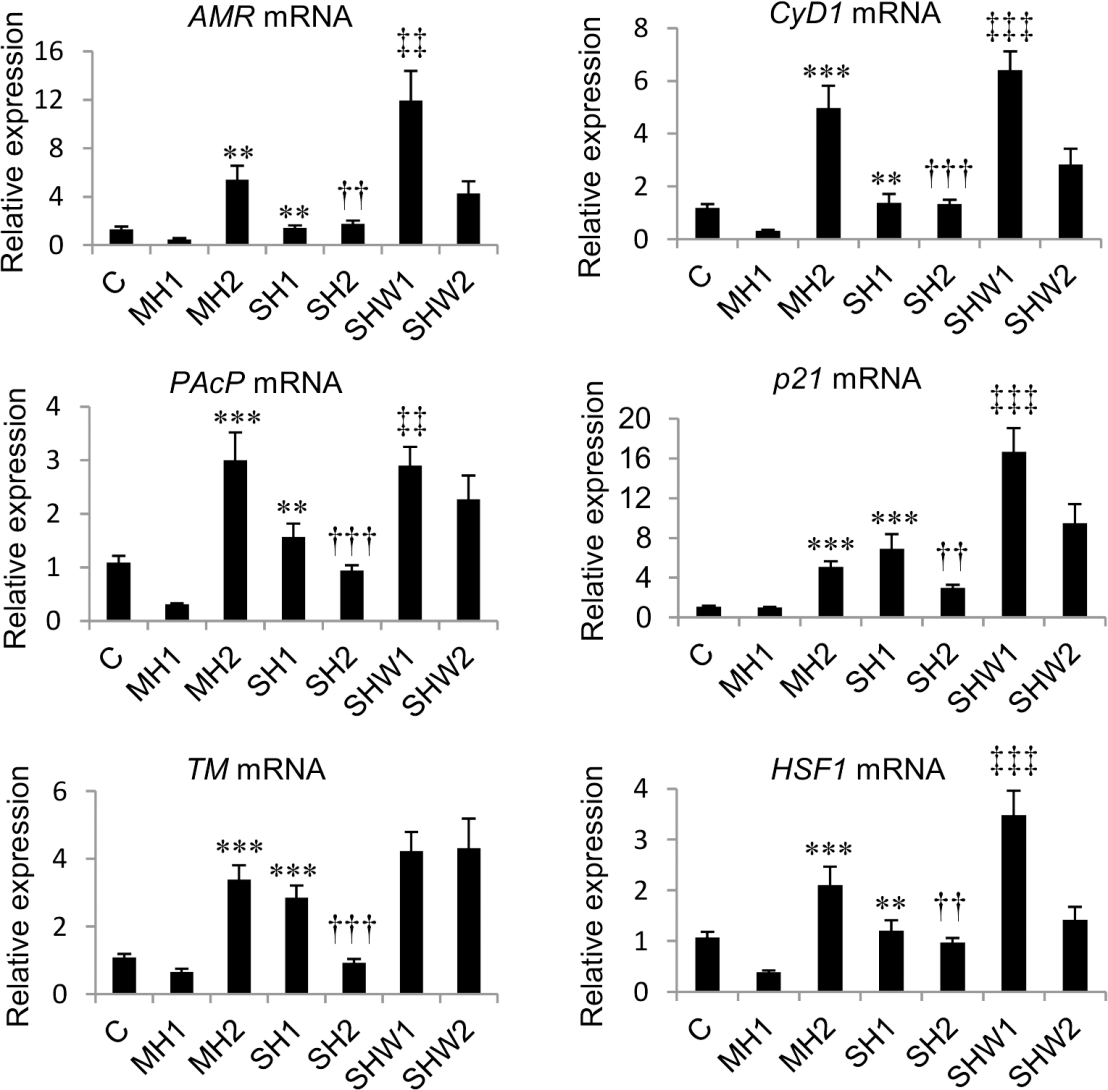
Relative mRNA expressions of *AMR*, *PAcP*, *TM*, *CyD*1, *p21* and *HSF1* in rat ventral prostate.

The mean relative mRNA expressions of *AMR*, *PAcP*, *TM*, *CyD1*, *p21* and *HSF1* and statistical data are presented in Fig 1 and Table 2.

**Table 2:** Statistically significant differences of relative mRNA expressions between control and other groups.

Gene symbol	C vs. MH1	C vs. MH2	C vs. SH1	C vs. SH2	C vs. SHW1	C vs. SHW2
*AMR*	*p* = 0.004	*p* = 0.001			*p* = 0.001	*p* = 0.008
*Bax*	*p* = 0.048	*p* = 0.000	*p* = 0.000		*p* = 0.000	*p* = 0.000
*Bcl-2*		*p* = 0.000	*p* = 0.000		*p* = 0.000	*p* = 0.000
*Bax/Bcl-2*		*p* = 0.038			*p* = 0.036	
*CyD1*	*p* = 0.000	*p* = 0.000			*p* = 0.000	*p* = 0.012
*HSF1*	*p* = 0.000	*p* = 0.008			*p* = 0.000	
*PAcP*	*p* = 0.000	*p* = 0.000			*p* = 0.000	*p* = 0.005
*p21*		*p* = 0.000	*p* = 0.000	*p* = 0.000	*p* = 0.000	*p* = 0.000
*TM*	*p* = 0.035	*p* = 0.000	*p* = 0.000		*p* = 0.000	*p* = 0.000

### *Bax* mRNA to *Bcl-2* mRNA ratio

The mean relative mRNA expressions of *Bax* were: C, 1.0 ± 0.1, MH1, 1.4 ± 0.2; MH2, 2.7 ± 0.4; SH1, 3.6 ± 0.8; SH2, 0.9 ± 0.1; SHW1, 3.0 ± 0.3, SHW2, 5.0 ± 1.0. The relative expression of *Bax* mRNA was higher in SH1 than in MH1 (*p* = 0.008) and lower in SH2 than in MH2 (*p* = 0.000). Statistically significant differences between group C and the other groups are presented in Table 2.

The mean relative mRNA expressions of *Bcl-2* were: C, 1.1 ± 0.1; MH1, 1.2 ± 0.3; MH2, 3.5 ± 0.5; SH1, 3.5 ± 0.7; SH2, 1.1 ± 0.2; SHW1, 4.7 ± 0.7 and SHW2, 4.9 ± 1.1. The relative expression of *Bcl-2* mRNA was higher in SH1 than in MH1 (*p* = 0.011) and lower in SH2 than in MH2 (*p* = 0.000). Statistically significant differences between group C and the other groups are presented in Table 2.

**Fig 2:**
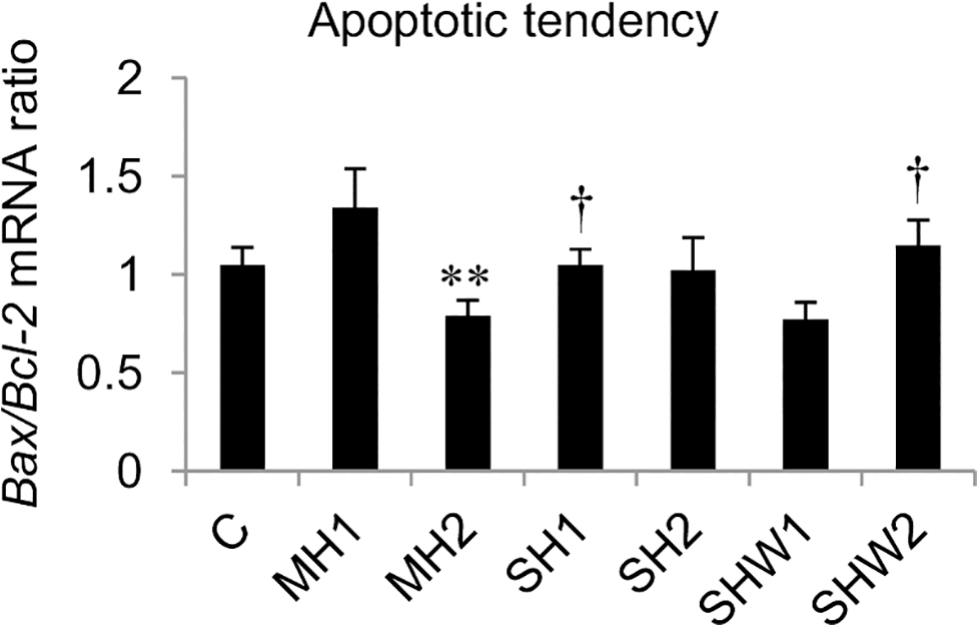
*Bax* mRNA to *Bcl-2* mRNA ratios in rat ventral prostate. *Bax/Bcl-2* mRNA ratio in MH1 correlated negatively with *AMR* mRNA (*r* = -0.848, *p* = 0.033).

The mean *Bax*/*Bcl-2* mRNA ratios are shown in Fig 2. Statistical data concerning group C comparisons are presented in Table 2. The mean relative expression of *Bax* mRNA exceeded the relative expression of *Bcl-2* mRNA in MH1 group, whereas the relative expression of *Bcl-2* mRNA exceeded the relative expression of *Bax* mRNA in MH2 and in SHW1.

### Amphiregulin protein expression in rat prostate ventral lobe

**Fig 3:**
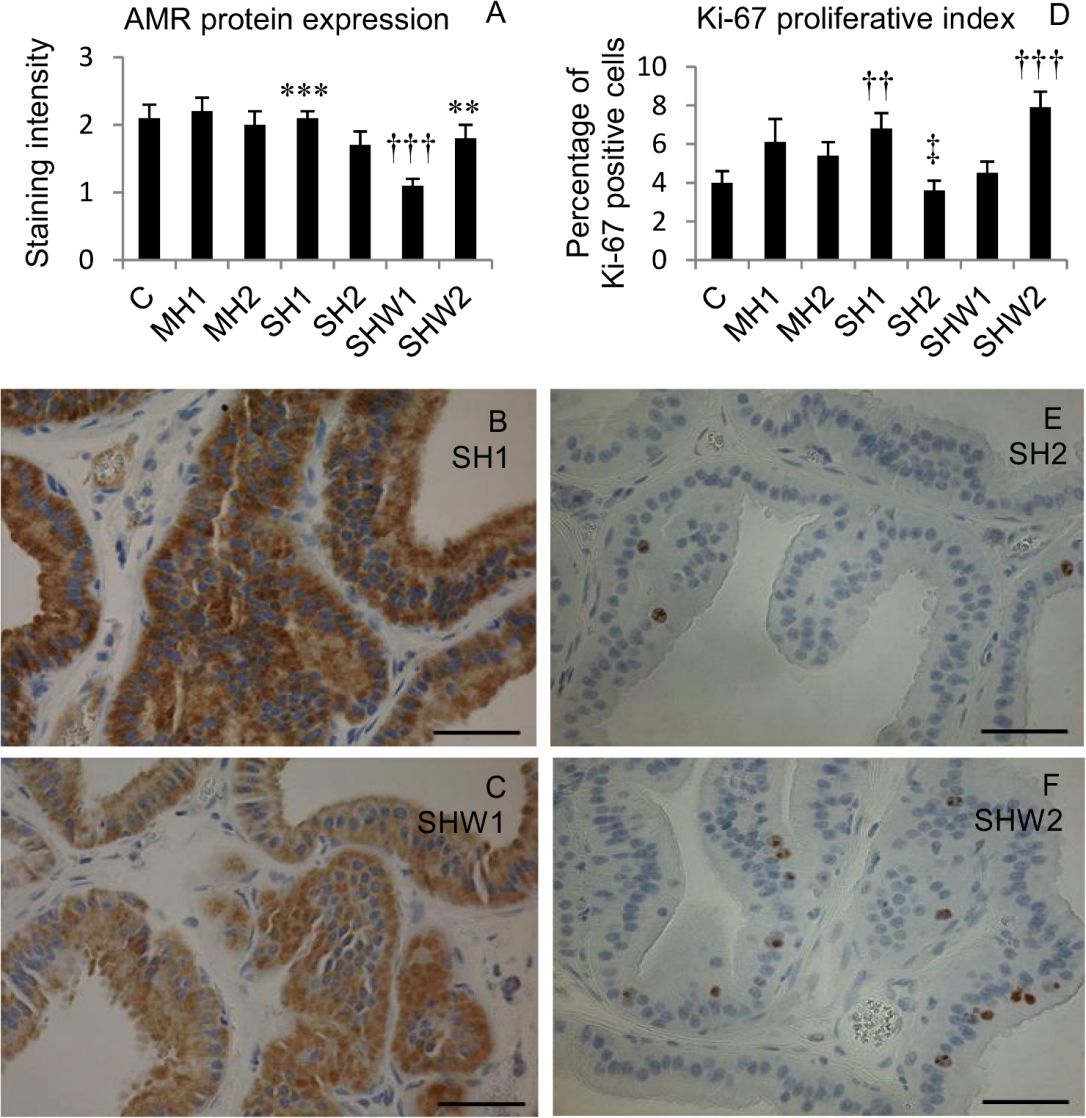
EGFR-ligand AMR protein expression and proliferative Ki-67 index in rat ventral prostate.

Cytoplasmic staining of luminal epithelial cells in rat ventral prostate lobe was detected in all of the groups. The mean values of AMR staining intensities and the statistically significant differences between rational pairs are presented in Fig 3A. The staining intensities in C, MH1, MH2 and SH1 groups were comparable to each other. The moderate cytoplasmic AMR staining in SH1 (Fig 3B) and in C was significantly higher than the weak cytoplasmic AMR staining in SHW1 (Fig 3C).

### Ki-67 proliferative index

Ki-67 positive nuclei could be seen in a proportion of epithelial cells in rat prostate ventral lobes. The mean proliferative index values as well as the statistically significant differences between rational pairs are presented in Fig 3D. Representative examples of the lowest percentage of Ki-67 positive nuclei in SH2 and the highest percentage of Ki-67 positive nuclei in SHW2 can be seen in Fig 3E and Fig 3F, respectively.

Ki-67 index correlated negatively with the relative *p21* mRNA expression in MH2 (*r* = -0.559, *p* = 0.030).

## Discussion and Conclusions

This study gives molecular level information about the effects of hypothermia and rewarming using a normal, healthy tissue as a model. Here we show that after an initial fall in gene expressions in MH1, 4.5-hour anesthesia-induced mild hypothermia at room temperature (MH2) and a shorter 2-hour cold exposure at +10°C (SH1) are sufficient to cause activated expression of pro-proliferative genes *AMR* and *CyD1* (Fig 1). Proliferative index Ki-67 was increased in SH1, and it reduced to significantly lower value in SH2 group with longer cold exposure and lower endpoint body temperature (Fig 3D). When the rats were warmed after 2-hour cold exposure (SHW1), depletion of AMR protein (Fig 3A) together with compensatory induction of *AMR* mRNA expression (Fig 1) could be seen. Ki-67 index rose to its highest value in SHW2 (Fig 3D), where normal body temperature was restored during rewarming.

Tissue homeostasis with balanced growth and cell death is compromised by cellular stress. Imbalanced relations between pro-apoptotic and anti-apoptotic signaling can be determined by the ratio of *Bax* and *Bcl-2*, two functionally antagonistic members of the same gene family. Higher *Bcl-2* mRNA expression appears to counterbalance the tendency to cell death, and higher *Bax* mRNA expression suggests a pro-apoptotic stimulus [22]. In the present study the shorter anesthesia-induced mild hypothermia in MH1 group caused a drop in *AMR* and *Cyd1* gene expressions (Fig 1, Table 2). In this group the expression of *Bax* exceeded the expression of *Bcl-2*, and *AMR* mRNA expression had a strong negative correlation with *Bax/Bcl-2* mRNA ratio (*r* = -0.848). This suggests favoring apoptosis in this group. Whereas in MH2 and SHW1, low *Bax/Bcl-2* mRNA ratio (Fig 2) together with highly expressed pro-proliferative *AMR* and *CyD1* mRNAs (Fig 1) obviously indicated that cells had entered a proliferative phase both in mild hypothermia with longer duration in MH2 and also in slower rewarming with body temperature staying clearly below normal in SHW1. At the time of total recovery of body temperature in SHW2, balanced ratio of *Bax* and *Bcl-2* (Fig 2, Table 2), but elevated Ki-67 index (Fig 3D) could be observed.

Initiation of prostatic diseases involves diverse factors. The etiology of chronic abacterial prostatitis/pelvic pain syndrome in humans is unknown, but it has been commonly connected with preceding event/events of cold exposure [23]. Association studies between prostatitis and prostate cancer have given controversial results, and causal relationship has been difficult to conclude [24]. When extreme cold is used in cryotherapy of prostate cancer, apoptosis of damaged cells is prominent at the warmer sub-freezing temperatures in the periphery of the treated area. Even if additional chemotherapy or radiotherapy is used to promote the existing susceptibility to apoptosis, recurrence of the disease is very frequent [25]. In this study, the cold exposure in prostate tissue was not comparable to temperatures in cryotherapy target area or in immediate proximity, but was likely to correspond to lowered temperatures further in the surroundings. When the rats recovered their normal body temperature (SHW2), apoptosis was at the level of the control group (Fig 2), while proliferation index in prostate tissue rose to its highest value (Fig 3D). Multiplication of cells after cold exposure appears not to be characteristic solely to remnant tumor cells, but concerns also normal, healthy cells.

As various stress conditions alter gene expressions, the function of a gene product might not always be the same. In a rodent model of lethal hemorrhage, profound hypothermia has been demonstrated to generally up-regulate gene expressions in pro-survival pathways and down-regulate in metabolic pathways [26]. In cultured mammalian cells it has been shown that mild hypothermia leads to activation of p21 and to a reversible G1/S cell cycle arrest [27, 28]. On the other hand, at low concentrations p21 is known to form a complex with CyD1 and cyclin dependent kinase 4, and thus p21 functions as a promoter of cell cycle progression [29]. Here, the relative expression of *p21* mRNA correlated negatively with proliferative Ki-67 index in MH2 (*r* = -0.559), which is consistent with the concept of concentration associated function of p21. Furthermore, induction of cell cycle arrest by p21 has been demonstrated to protect cells from apoptosis [16]. In some cell lines, however, increased expression of p21 has also been shown to promote apoptosis [30]. No correlations between *p21* mRNA and *Bax/Bcl-2* mRNA ratio could be detected in this study.

Gene expressions of *p21*, *PAcP*, *TM* and *HSF1* follow the double peaked expression pattern of *AMR* and *CyD1* (Fig 1). We have previously studied *TM* and *HSF1* gene expressions in rat cardiac tissue. Hypothermia-induced increases of *TM* and *HSF1* mRNA levels in heart were detected in SH1 group, instead of MH2 and SH1 in prostate in the present study. In addition, expressions of these genes in heart tissue stayed at the level of control in rewarming [13]. After nearly fatal 4-hour hypothermia with +15°C body temperatures it has been shown that the gene expressions of numerous genes in rat myocardium increase during the following rewarming [31]. Divergent mRNA expression profiles in heart and prostate tissues in our studies, with clearly milder cold exposure, reflect functional control of body temperature in vital and non-vital organs. Vital organs in mammals are protected in hypothermia by thermoregulatory vasoconstriction, which keeps metabolic heat in the core compartment, not allowing it to escape into peripheral tissues [32]. In spite of anesthesia-induced heat redistribution within the body [20], the temperature in heart is most likely higher than in prostate with the cold exposures used by us. Therefore the temperature of heart tissue in SH1 was probably closer to the temperature of prostate in MH2, and did not lower enough to cause the following increase in gene expressions in rewarming phase. The number of animals in this study is sufficient for reliable interpretation. This is, however, a rat model and proving its relevance to humans needs further research.

In conclusion, gene expression profiles in cold exposure and following rewarming appear to be organ-specific with some variations depending on the function of each gene. Hypothermia and following rewarming cause multiplication of cells in healthy rat prostate ventral lobe possibly via ErbB signaling pathway. The data gained from this study adds to the knowledge of molecular level effects of hypothermia in healthy organisms.
