# Correction: Hypothermia and Rewarming Induce Gene Expression and Multiplication of Cells in Healthy Rat Prostate Tissue

**DOI:** 10.1371/journal.pone.0131261

**Published:** 2015-06-12

**Authors:** The PLOS ONE Staff

The second sentence of the final paragraph of the Introduction is incorrect. The correct sentence is: Ventral lobes of rat prostate tissues were used in determination of the relative mRNA expressions of *AMR*, *TM* and the transmembrane form of the *PAcP* (Quintero et al. 2007). Cellular form of *PAcP* in the cytosol has been suggested to down-regulate prostate cell growth in human prostate cancer cells [15].

There are errors in Table 1, “Primers used in qPCR analysis.” Please see the corrected Table 1 here.

**Table 1:** Primers used in qPCR analysis

Gene symbol/ accession number	Direction	Primer sequence	Amplicon size
*AMR* NM_017123.1	Forward	GTGCATGCCATTGCCTAGCTGA	78
	Reverse	TCATTTCCGGTGTGGCTTGGCA	
*Bax* NM_017059.2	Forward	CCAGGACGCATCCACCAAGAAGC	136
	Reverse	TGCCACACGGAAGAAGACCTCTCG	
*Bcl-2* NM_016993.1	Forward	GAGGCTGGGATGCCTTTGTGGA	89
	Reverse	GCTGAGCAGCGTCTTCAGAGA	
*CyD1* NM_171992.4	Forward	ATCAAGTGTGACCCGGACTG	216
	Reverse	GCCACTACTTGGTGACTCCC	
*HSF1* XM_006241890.1	Forward	CCATGAAGCACGAGAACGAG	117
	Reverse	ACTGCACCAGTGAGATCAGGA	
*PAcP* NM_001134901.1	Forward	CGGGATCCTGGTGATATTGCT	70
	Reverse	CCGATACACGTCTCTCTGCC	
*p21* NM_080782.3	Forward	ACATCTCAGGGCCGAAAACG	78
	Reverse	CTTGCAGAAGACCAATCGGC	
*TM* NM_031771.2	Forward	GATCTCCATTGCCAGCCT	140
	Reverse	CACGTGCTGCAGTACTACCT	
*GAPDH* BC029618	Forward	TGGAAGGACTCATGACCACA	160
	Reverse	TTCAGCTCAGGGATGACCTT	

The legend for Fig 1, “Relative mRNA expressions of *AMR*, *PAcP*, *TM*, *CyD1*, *p21* and *HSF1* in rat ventral prostate,” does not appear. Please see the complete, correct Fig 1 legend here.

**Fig 1:**
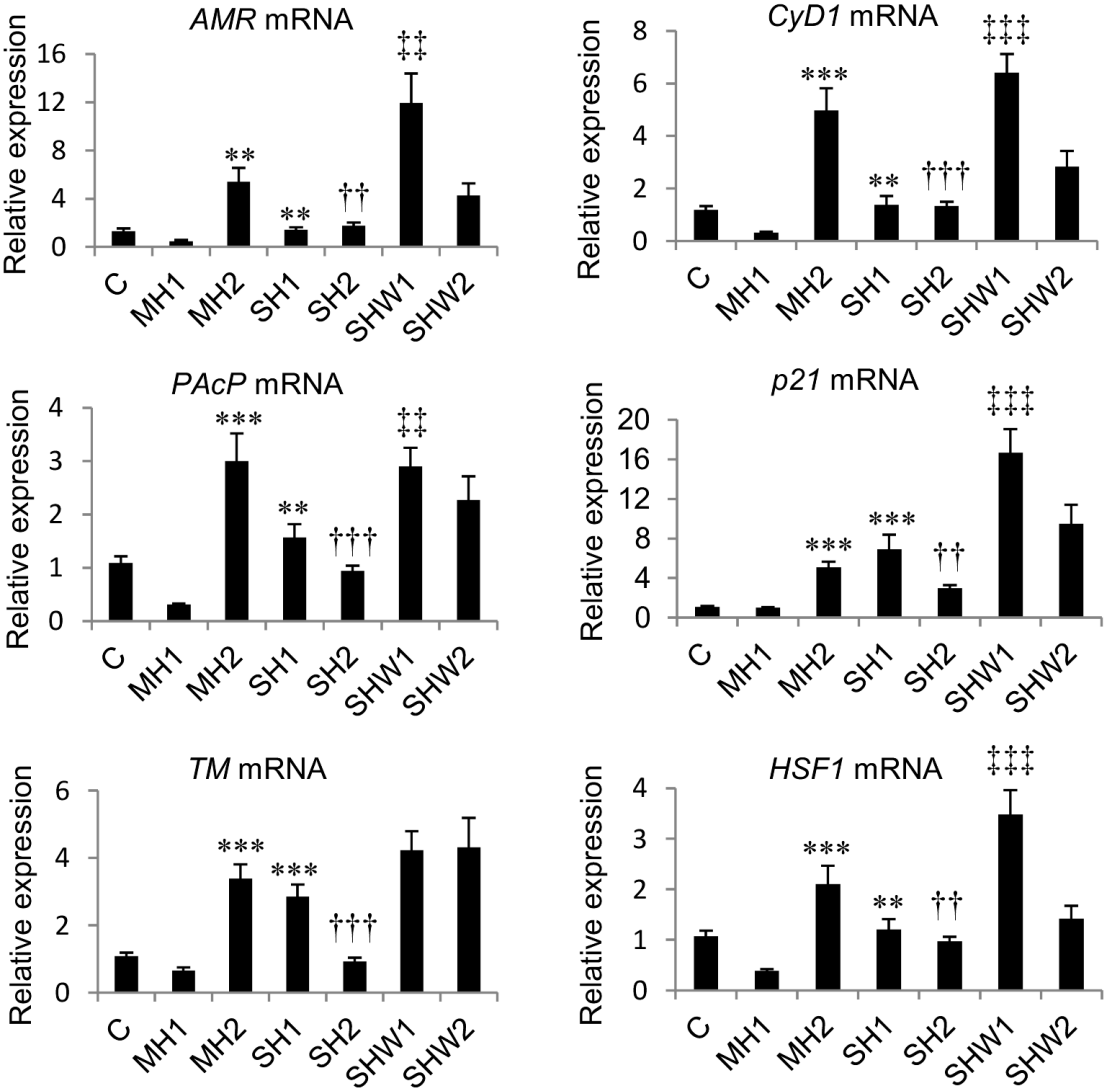
Relative mRNA expressions of *AMR*, *PAcP*, *TM*, *CyD1*, *p21* and *HSF1* in rat ventral prostate. C, control; MH1, mild hypothermia 1; MH2, mild hypothermia 2; SH1, severe hypothermia 1; SH2, severe hypothermia 2; SHW1, severe hypothermia followed by rewarming at room temperature; SHW2, severe hypothermia followed by rewarming at +28C; **p≤0.01, ***p≤0.001 compared with MH1; ††*p*≤0.01, †††*p*≤0.001 compared with MH2; ‡‡*p*≤0.01, ‡‡‡*p*≤0.001 compared with SH1; data are mean ± SEM.

The legend for Fig 2, “*Bax* mRNA to *Bcl-2* mRNA ratios in rat ventral prostate” does not appear. Please see the complete, correct Fig 2 caption here.

**Fig 2:**
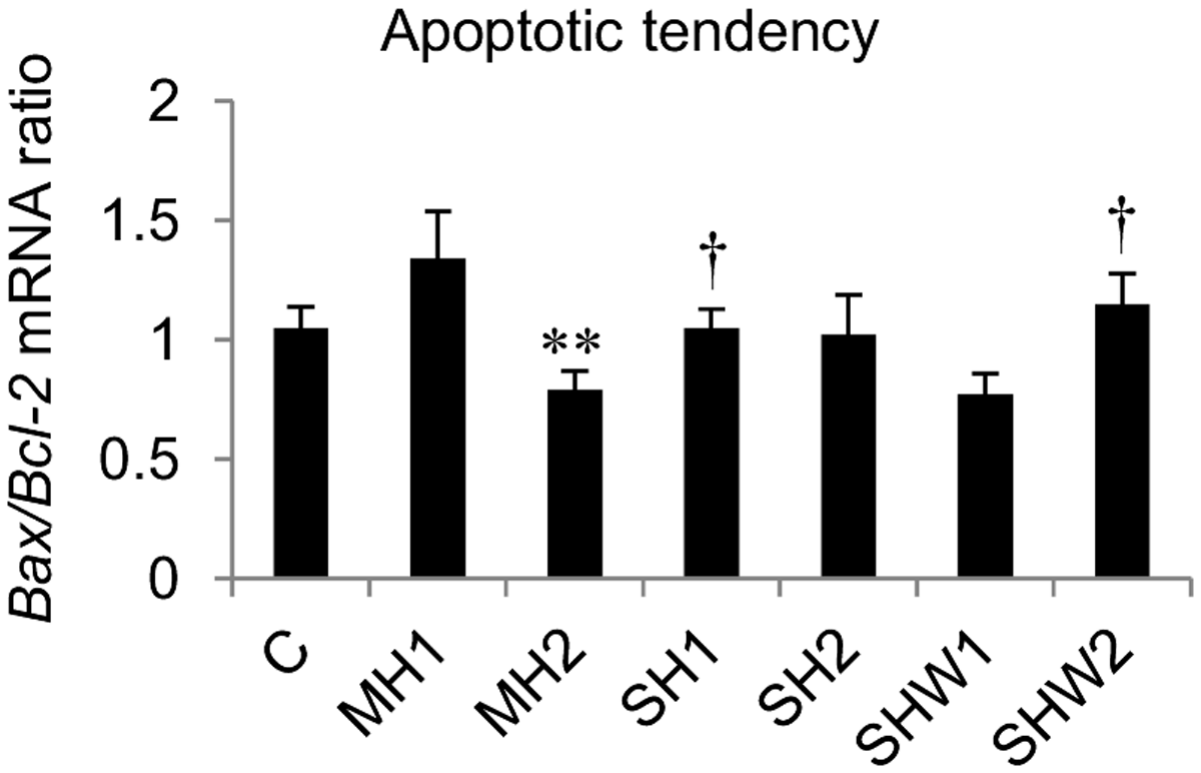
*Bax* mRNA to *Bcl-2* mRNA ratios in rat ventral prostate. C, control; MH1, mild hypothermia 1; MH2, mild hypothermia 2; SH1, severe hypothermia 1; SH2, severe hypothermia 2; SHW1, severe hypothermia followed by rewarming at room temperature; SHW2, severe hypothermia followed by rewarming at +28C; **p≤0.01 compared with MH1; †*p*≤0.05 compared with SHW1; data are mean ± SEM.

The legend for Fig 3, “EGFR-ligand AMR protein expression and proliferative Ki-67 index in rat ventral prostate” does not appear. Please see the complete, correct Fig 3 caption here. The publisher apologizes for these errors.

**Fig 3:**
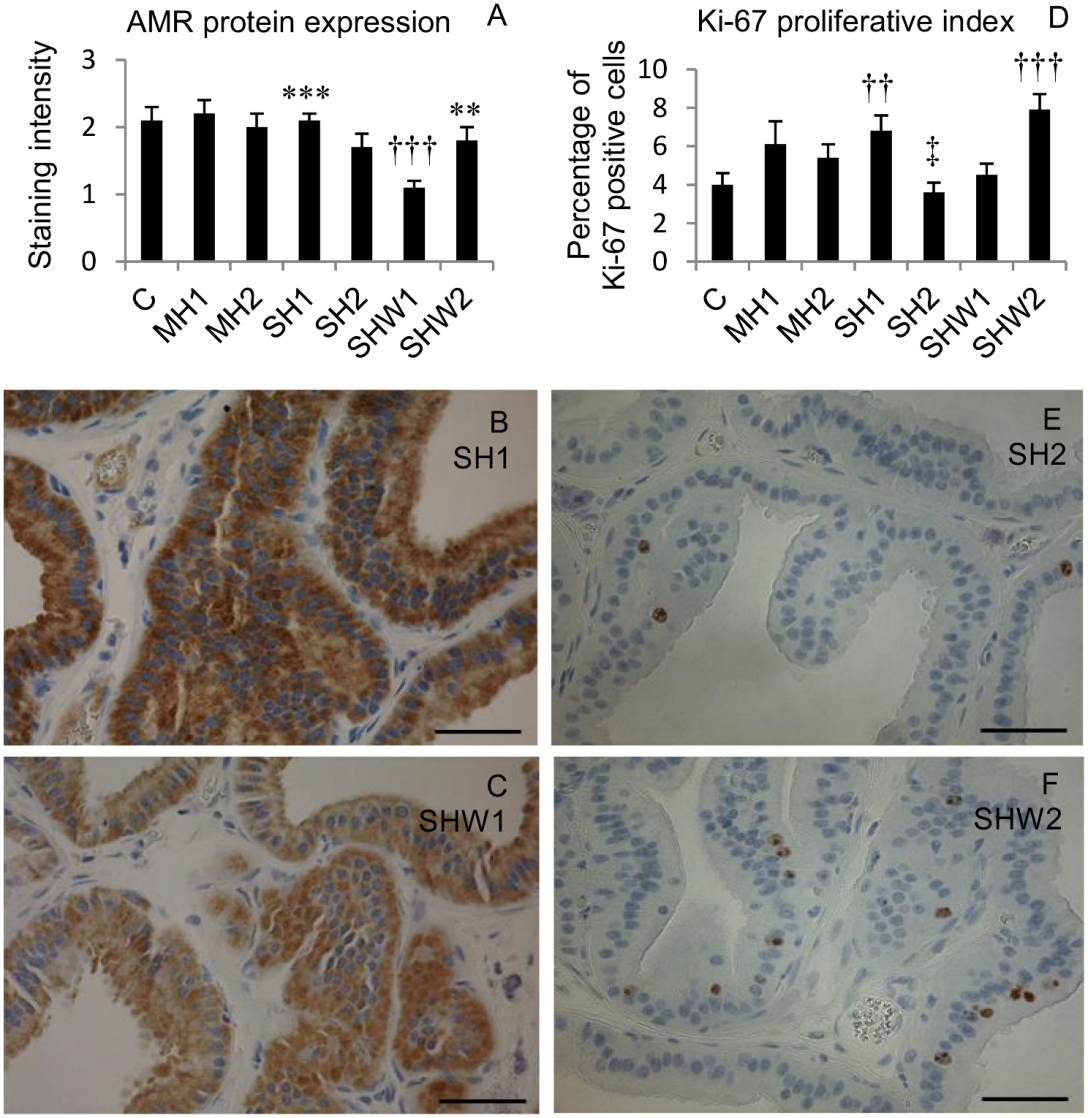
EGFR-ligand AMR protein expression and proliferative Ki-67 index in rat ventral prostate. Mean AMR protein expression values in rat prostate ventral lobe tissues (A); cytosolic AMR immunoreactivity in SH1 (B) and in SHW1 (C); mean Ki-67 index values in rat prostate ventral lobe tissues (D); nuclear Ki-67 immunoreactivity in SH2 (E) and in SHW2 (F). C, control; MH1, mild hypothermia 1; MH2, mild hypothermia 2; SH1, severe hypothermia 1; SH2, severe hypothermia 2; SHW1, severe hypothermia followed by rewarming at room temperature; SHW2, severe hypothermia followed by rewarming at +28C; **p≤0.01, ***p≤0.001 compared with SHW1; ††*p*≤0.01, †††*p*≤0.001 compared with C; ‡*p*≤0.05 compared with MH2; scale bar = 50 μm; data are mean ± SEM.
