# Correction: Correction: Hypothermia and Rewarming Induce Gene Expression and Multiplication of Cells in Healthy Rat Prostate Tissue

**DOI:** 10.1371/journal.pone.0137228

**Published:** 2015-08-25

**Authors:** The PLOS ONE Staff

A reference is omitted from the originally published article as well as the correction published on June 12, 2015. In the correction the reference is cited as Quintero et al. 2007.

The full reference is: Quintero IB, Araujo CL, Pulkka AE, Wirkkala RS, Herrala AM, Eskelinen EL, Jokitalo E, Hellström PA, Tuominen HJ, Hirvikoski PP, Vihko PT (2007) Prostatic acid phosphatase is not a prostate specific target. Cancer Res. 67(14): 6549–54.

The publisher apologizes for the error.
